# Exposure to *Aedes aegypti* Bites Induces a Mixed-Type Allergic Response following Salivary Antigens Challenge in Mice

**DOI:** 10.1371/journal.pone.0155454

**Published:** 2016-05-20

**Authors:** Michele S. Barros, Eliane Gomes, Daniele I. Gueroni, Anderson D. Ramos, Luciana Mirotti, Esther Florsheim, Bruna Bizzarro, Ciro N. R. Lino, Ceres Maciel, Adriana Lino-Dos-Santos-Franco, Wothan Tavares-de-Lima, Margareth L. Capurro, Momtchilo Russo, Anderson Sá-Nunes

**Affiliations:** 1 Department of Immunology, Institute of Biomedical Sciences, University of Sao Paulo, Sao Paulo - SP, Brazil; 2 Institute of Immunology, Hannover Medical School, Hannover, Germany; 3 Graduate Program in Biophotonics Applied to Health Sciences, University Nove de Julho (UNINOVE), São Paulo - SP, Brazil; 4 Department of Pharmacology, Institute of Biomedical Sciences, University of Sao Paulo, Sao Paulo - SP, Brazil; 5 Department of Parasitology, Institute of Biomedical Sciences, University of Sao Paulo, Sao Paulo - SP, Brazil; 6 National Institute of Science and Technology in Molecular Entomology, National Council of Scientific and Technological Development (INCT-EM/CNPq), Rio de Janeiro - RJ, Brazil; Onderstepoort Veterinary Institute, SOUTH AFRICA

## Abstract

Classical studies have shown that *Aedes aegypti* salivary secretion is responsible for the sensitization to mosquito bites and many of the components present in saliva are immunogenic and capable of inducing an intense immune response. Therefore, we have characterized a murine model of adjuvant-free systemic allergy induced by natural exposure to mosquito bites. BALB/c mice were sensitized by exposure to *A*. *aegypti* mosquito bites and intranasally challenged with phosphate-buffered saline only or the mosquito’s salivary gland extract (SGE). Blood, bronchoalveolar lavage (BAL) and lung were collected and evaluated for cellularity, histopathological analyses, cytokines and antibody determination. Respiratory pattern was analyzed by Penh measurements and tracheal segments were obtained to study *in vitro* reactivity to methacholine. BAL recovered from sensitized mice following challenge with SGE showed an increased number of eosinophils and Th2 cytokines such as IL-4, IL-5 and IL-13. Peribronchoalveolar eosinophil infiltration, mucus and collagen were also observed in lung parenchyma of sensitized mice, suggesting the development of a typical Th2 response. However, the antibody profile in serum of these mice evidenced a mixed-type response with presence of both, IgG1/IgE (Th2-related) and IgG2a (Th1-related) isotypes. In addition, changes in breathing pattern and tracheal reactivity to methacholine were not found. Taken together, our results show that *A*. *aegypti* bites trigger an atypical allergic reaction, with some classical cellular and soluble Th2 components in the lung, but also systemic Th1 and Th2 antibody isotypes and no change in either the respiratory pattern or the trachea responsiveness to agonist.

## Introduction

*Aedes aegypti* is one of the most well characterized mosquito species in public health and the primary vector of arbovirus causing important diseases such as dengue fever, yellow fever, Chikungunya fever, and Zika fever in tropical areas [1,2]. *A*. *aegypti* females require a blood meal for ovary maturation and optimal development of their eggs [3]. The salivary secretion injected into the host skin during the blood feeding is rich in pharmacologically active substances that allow the mosquito to successfully feed by counteracting host hemostatic, inflammatory and immunological defenses [3–6], and it also provides an essential vehicle for pathogen transmission to vertebrate hosts [7]. Furthermore, salivary allergens are related to local cutaneous reactions and, in some cases, to systemic responses in atopic individuals [8,9].

Allergy to mosquito bites is common and of increasing clinical significance, as it may impair the quality of life for many people around the world [8,10,11]. Classical works have demonstrated that salivary secretion is responsible for sensitization to mosquito bites and the salivary antigens are involved in the elicitation of immediate and delayed skin reactions to bites [12,13]. Mosquitoes that have had their main salivary duct cut are able to get a blood meal from a bite-sensitized individual, but the bite does not cause cutaneous reactions as those observed for normal mosquitos [14]. Although several allergens have been identified in mosquito whole extract, just a few present in the salivary glands of *A*. *aegypti* females have known functions in allergic response, namely Aed a 1 [15], Aed a 2 [16] and Aed a 3 [17]. All these allergens have been recognized by the IgE produced in mosquito-allergic subjects.

Animal models provide a valuable resource for investigating disease mechanisms and progression. In the case of a complex multifactorial allergic disease such as asthma, a single animal model rarely reproduces all of the morphological and functional features of the chronic human disease [18]. One advantage of natural sensitization is the absence of adjuvant use, mimicking the allergy that the majority of the population develops during its lifetime. In this sense, the natural sensitization model to mosquito bites can be a great tool to study multiple parameters of the immune response induced by saliva, while it can also be used to further study allergic diseases and mechanisms involved in sensitization and desensitization to allergens. Here, we characterize a mouse model of allergy using natural exposure to mosquito bites and posterior intranasal challenge with *A*. *aegypti* salivary antigens. Local and systemic responses against these salivary compounds were evaluated through the analysis of pulmonary cell infiltration, total IgE, antigen-specific IgG1 and IgG2a, respiratory pattern, trachea responsiveness, mucus production, collagen deposition, and Th1 and Th2 cytokine production.

## Materials and Methods

All experiments involving laboratory animals were evaluated by the “Ethics Committee for Animal Use” from the Institute of Biomedical Sciences—University of São Paulo (our Institutional Animal Care and Use Committee) and approved under the protocol numbers 140/2011 and 148/2011. The procedures are according to the Brazilian National Law number 11794 from 10/08/2008, which regulates all research activities involving animal use in the country. Anesthesia was performed prior to mosquito exposure and to sensitization with SGE plus aluminum hydroxide (ketamine 50 mg/kg plus xylazine 20 mg/kg i.p.). Euthanasia was performed for in vitro trachea responsiveness measurements (sodium pentobarbital 360 mg/kg i.p.) and for blood, BAL and blood collection, and lung removal (halothane inhalation). None of the animals used in the present work became ill or died prior to the experimental endpoint.

### Mice

BALB/c mice (female, 6–8 weeks old) were bred and maintained at the Isogenic Breeding Unit (Department of Immunology, Institute of Biomedical Sciences, University of São Paulo, Brazil) under specific pathogen-free conditions. For the experiments, animals were divided into groups containing 6–8 mice.

### Mosquitoes and Salivary Gland Extract (SGE)

*A*. *aegypti* mosquitoes (male and female) were bred in an insectary at the Department of Parasitology, ICB/USP, Brazil, where they were fed and mated as previously described [19]. Four- to eight-day-old female adult mosquitoes were used for the experimental mice sensitization and salivary gland extract (SGE) preparation as described by Bizzarro *et al*. [20].

### Mice Sensitization by Mosquito Bites

During the sensitization protocol, BALB/c mice were separated into two groups and identified according to the number of exposures to mosquito bites (1x or 4x). Then, each mouse was anesthetized with ketamine (50 mg/kg) and xylazine (20 mg/kg) intraperitoneally (i.p.) and placed on the top of a cylindrical transparent plastic container (12 cm diameter) covered with tulle fabric and containing 50 female mosquitoes, for 30 min. This procedure was performed one or four times using a 14-day interval (days 0, 14, 28 and 42) and the proportion of mosquitoes fed at each sensitization was typically ≥ 80%. For the induction of lung inflammation, mice from 1x and 4x groups received two intranasal (i.n.) challenges of 10 μg SGE in 50 μL of sterile PBS at days 49 and 56. Control groups of non-sensitized mice consisted of: a) two i.n. challenges with 10 μg SGE in 50 μL of sterile PBS (SGE group) or; b) two i.n. challenges with 50 μL of sterile PBS (PSB group) at the same days, as described above. Both challenges were performed under anesthesia with ketamine and xylazine, as described above. Twenty-four hours after the first challenge (day 50), the respiratory pattern analysis was performed and 24 h after the second challenge (day 57), blood, BAL, tracheal segments, and lung were collected to study *in vitro* reactivity to methacholine.

### Mice Sensitization with SGE and Aluminum Hydroxide

One group of BALB/c mice was naturally immunized by repeated exposures to approximately 20 female mosquitoes as described above, and another group was sensitized by inoculation with SGE (20 μg/animal) adsorbed in 1.6 mg of aluminum hydroxide gel (Alum) subcutaneously (s.c.), which maximizes IgE production. This procedure was repeated 4 times at a 15-day interval under anesthesia with ketamine and xylazine. A control group of the same age was either not exposed to mosquito bites or sensitized with SGE/Alum. Blood samples were collected 15 days after each immunization protocol and serum samples were separated and stored at -80°C until use for serum antibody level determinations.

### Determination of Respiratory Pattern

Respiratory parameters were evaluated 24 h after the first challenge. Changes in breathing patterns were determined before and after the administration of increasing doses of inhaled methacholine (12 and 25 mg/mL) by a noninvasive method in conscious and unrestrained mice using a single-chamber, whole-body plethysmograph (Buxco Electronics Inc., Wilmington, NC, USA). The respiratory patterns were recorded 5 minutes after nebulization with PBS and again after nebulization with methacholine. Dose-response curves to methacholine were calculated as the average of the values of enhanced pause (Pehn). Pehn is a dimensionless value that represents a function of the ratio of peak expiratory flow to peak inspiratory flow and a function of the timing of expiration. The technique has been previously validated in animal models of airway responsiveness [21].

### Tracheal *In Vitro* Responsiveness to Methacholine

Twenty-four hours after the last challenge (day 57), mice received an overdose of anesthetic (sodium pentobarbital; > 360 mg/kg, i.p.). Three to five tracheal rings were collected from the area closest to the larynx and placed in 15 ml organ chambers immersed in Krebs–Henseleit buffer (95% O_2_ and 5% CO_2_) at 37°C. Responsiveness was measured as isometric tension induced by a cumulative doses of methacholine (MCh, 10^−9^ to 10^−3^ M) [22] using a force displacement transducer coupled to a chart recorder (Powerlab^®^, Labchart, AD Instruments). Assessment of tissue viability was performed by replacing Krebs–Henseleit buffer by depolarizing KCl buffer (60 mM).

### Blood Collection

Twenty-four hours after the last challenge (day 57), mice were deeply anesthetized with halothane and blood samples were collected through cardiac puncture. Serum was separated and stored at −80°C until use for antibody level determinations.

### BAL Collection and Cell Counting

After blood collection, the trachea was cannulated with a polyethylene tube (1-mm inner diameter) and the lung was washed once by flushing with 1 mL PBS. The recovered BAL was centrifuged (400 *g*, 10 min, 4°C); the supernatant was stored at -80°C until use for cytokine level determination and the resulting cell pellet was then resuspended in 1 mL PBS. Aliquots of the cell suspension were diluted with Turk solution (v/v) and the total cell number was determined in Neubauer chambers. The differential cell count was performed in cytocentrifuge preparations (BIO Research, São Paulo, SP, Brazil) and stained with the hematological dye “3 Step Staining Set” (Richard-Allan Scientific, Kalamazoo, MI, USA) under lens oil immersion with the objective to determine the percentage of macrophages, lymphocytes, neutrophils, and eosinophils.

### Lung Histology

After BAL collection, lungs were perfused via the heart right ventricle with 10 mL of cold PBS to remove residual blood. The largest lobe of the left lung was removed and immersed in 10% phosphate-buffered formalin for 24 h to preserve the pulmonary architecture, followed by 70% ethanol and then, embedded in paraffin. Tissue sections of 5-μm were then stained with haematoxylin/eosin (H&E), periodic acid-Schiff (PAS) and Masson's trichrome staining for the analysis of cellular infiltration, mucus production, and collagen deposition in lung tissues, respectively. A quantitative digital morphometric analysis was performed using the program Metamorph 6.0 (Universal Images Corp. Downingtown, PA, USA). Circumference areas of the bronchi or lung parenchyma were measured electronically and mucus and collagen indexes were determined as the percentage of the area stained with PAS and Masson's trichrome staining, respectively.

### Flow Cytometry

Cells isolated from lung tissue after collagenase and deoxyribonuclease (DNAse) digestion or from BAL were stained with florescence-conjugated antibodies for cell surface markers MHC class II, Gr1 (Ly-6G), Siglec F, CD4, CD8, and CD19 (BD Biosciences) for 30 minutes at 4°C in the dark. After the wash, cells were transferred to polypropylene tubes (12 x 75 mm) and acquired by a FACSCanto II flow cytometer (BD Biosciences). Data was analyzed using the FlowJo software, version 7.5.5 (Tree Star, Ashland, OR, USA).

### Cytokine and Antibody Determinations

The levels of the cytokines IL-4, IL-5, IL-10 and IFN-γ in the BAL were assayed by OptEIA^™^ ELISA sets (BD Biosciences, San Diego, CA, USA); the levels of IL-13 were evaluated by DuoSet ELISA (R&D Systems, Minneapolis, MN, USA), and those for IL-17 by ELISA MAX (Biolegend, San Diego, CA, USA), according to the manufacturers’ recommendations. Values were expressed as pg/mL and deduced from standard curves of recombinant cytokines.

Measurement of total immunoglobulin E (IgE) was assayed by OptEIA^™^ ELISA sets according to the manufacturer's recommendation (BD Biosciences). For specific IgG1 and IgG2a antibodies, an ELISA was standardized in our lab for this purpose. Briefly, plates were coated overnight at 4°C with SGE (500 ng/well/100 μL) or OVA (2 μg/well/100 μL). After blocking the wells with PBS/SFB 10%, serum samples were added and bound IgG1 and IgG2a were revealed with a peroxidase-labelled antibody (BD Bioscience and Invitrogen, respectively).

## Statistical Analysis

Statistical analyses of differences between means of experimental groups were performed using Student’s *t* test (for comparison of two groups) or analysis of variance (ANOVA) followed by Tukey as a post-test (for three or more groups). A value of *p* less than or equal to 0.05 was considered statistically significant. Data are expressed as mean plus the standard error of the mean (SEM).

## Results

### Exposure to *A*. *aegypti* Bites followed by Challenge with SGE Induces an Inflammatory Infiltrate to the BAL with Predominance of Eosinophils and Lymphocytes

**Fig 1:**
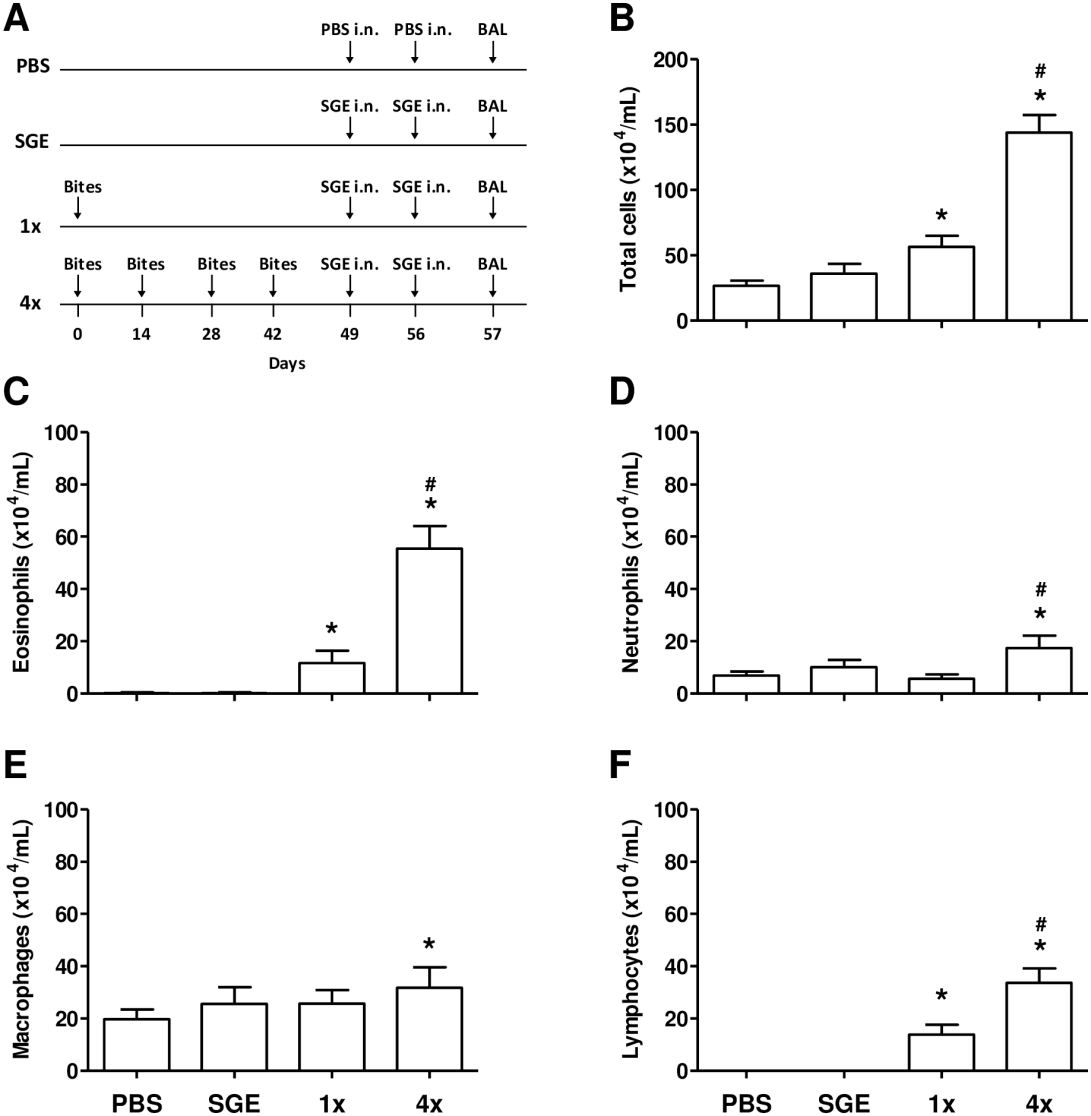
Mice sensitized by mosquito bites develop eosinophilic airway inflammation after intranasal SGE challenge. BAL collection from control and sensitized groups was performed 24 h after the last challenge with PBS or SGE. Total cell number and differential cell counts were analyzed by microscopic evaluation of cytocentrifuged slides. **(A)** Sensitization protocol; **(B)** total cells; **(C)** eosinophils; **(D)** neutrophils; **(E)** macrophages; **(F)** lymphocytes. Results were expressed as mean ± SEM (n = 6). *p < 0.05 when compared with PBS group; ^*#*^*p* < 0.05 when compared with the 1x group.

BAL cells were analyzed to evaluate inflammatory changes within the lung of BALB/c mice sensitized or not to mosquito bites and intranasally challenged with PBS or SGE (experimental protocol in Fig 1A). Under our experimental conditions, no significant cellular recruitment occurred when non-sensitized mice received SGE challenge in comparison to the animals of the control group (PBS-challenged mice). However, mice naturally exposed to mosquitoes once (1x group) or four times (4x group) and challenged with SGE demonstrated a significant increase in the recruitment of inflammatory cells to the airways compared with the control group (PBS-challenged mice), as observed by the total number of cells recovered from the BAL (Fig 1B). Moreover, the comparison between both sensitized groups showed that the inflammatory infiltrate was significantly higher in the 4x group when compared to the 1x group (Fig 1B). Differential cell count analysis demonstrated that eosinophils were the predominant cell type present in the inflammatory infiltrate and the increase observed for both groups, 1x and 4x, was significantly different when compared to the control group. In addition, eosinophil numbers in the BAL of the 4x group were significantly higher than those found in the BAL of the 1x group (Fig 1C). For neutrophils and macrophages (Fig 1D and 1E, respectively), only the 4x group presented a slightly significant increase compared to the control group. Likewise, in lymphocyte analysis, the increase seen in the 4x group was statistically significant in relation to PBS and the 1x group (Fig 1F).

**Fig 2:**
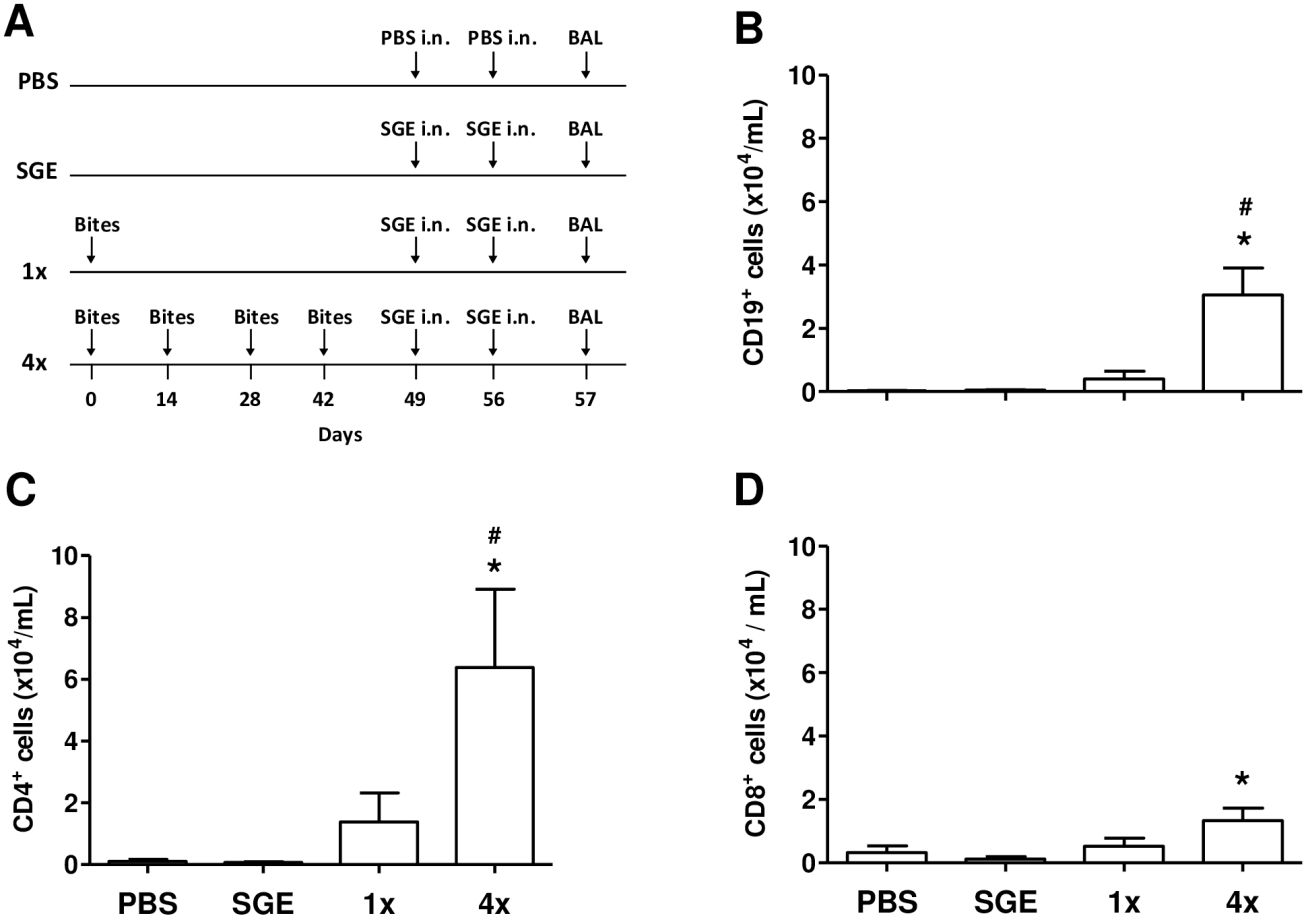
B and T cells are increased in the BAL of mice sensitized by mosquito bites followed by SGE challenge. BAL collection from control and sensitized groups was performed 24 h after the last challenge with PBS or SGE. Differential analyses of lymphocytes were performed by flow cytometry in cells stained with florescence-conjugated antibodies for cell surface markers. **(A)** Sensitization protocol; **(B)** total number of CD19^+^ cells, **(C)** total number of CD4^+^ cells; **(D)** total number of CD8^+^ cells. Results were expressed as mean ± SEM (n = 6). *p < 0.05 when compared with PBS group; ^*#*^*p* < 0.05 when compared with the 1x group.

In order to characterize the lymphocyte populations present in the BAL, cells were analyzed by flow cytometry (experimental protocol in Fig 2A). From those, B cells (CD19^+^—Fig 2B) and helper T cells (CD4^+^—Fig 2C) were all significantly increased in the BAL of group 4x when compared to the control group (PBS-challenged mice). Similarly, there was a slight increase in CD19^+^ and CD4^+^ cells in the BAL of group 1x compared to the control group, however, the difference did not reach statistical significance (Fig 2B and 2C, respectively). Of note, the number of CD8^+^ T cells detected in the BAL of all groups was very low, but it was significantly increased in the 4x group when compared to the control group (Fig 2D). Since the 4x group displayed the most significant changes in the experimental model employed, the 1x group was not included in the subsequent assays.

### Exposure to *A*. *aegypti* Bites followed by SGE Challenge Induces the Production of Th2-type, but Not Th1- or Th17-type Cytokines in the BAL

**Fig 3:**
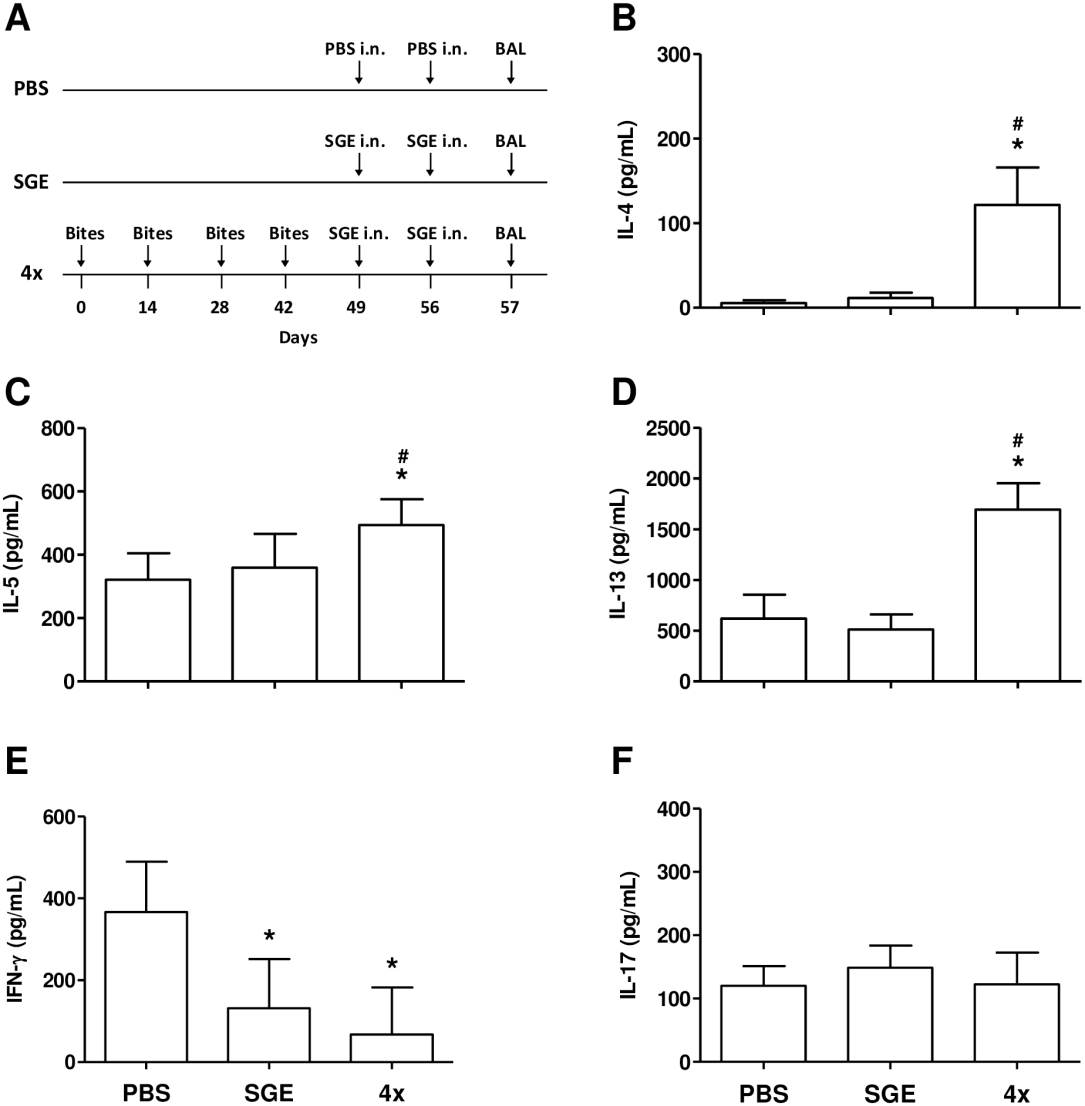
Th2 cytokine levels are upregulated in BAL of sensitized mice in response to SGE challenge. BAL of control and sensitized mice was collected 24 h after the last challenge with PBS or SGE, and the cytokine levels in the free-cell supernatant were determined by ELISA. **(A)** Sensitization protocol; **(B)** IL-4; **(C)** IL-5; **(D)** IL-13; **(E)** IFN-γ; **(F)** IL-17. Results were expressed as mean ± SEM (n = 6). *p < 0.05 compared with PBS group; ^*#*^*p* < 0.05 compared with SGE group.

Because allergic airway inflammation is considered a Th2-driven phenomenon [23], we next determined the cytokine profile present in the BAL (experimental protocol in Fig 3A). In fact, a significant increase in the Th2 cytokines IL-4 (Fig 3B), IL-5 (Fig 3C) and IL-13 (Fig 3D) levels was observed in BAL supernatant of 4x group compared to PBS- or SGE-challenged groups. On the other hand, IFN-γ (a Th1 cytokine) levels were decreased in BAL of both SGE-challenged and 4x groups when compared to PBS group (Fig 3E). No significant differences in IL-17 (a Th17 cytokine) levels were detected among the groups (Fig 3F).

### Eosinophils, Mucus and Collagen Selectively Accumulate in Lung Parenchyma upon Exposure to *A*. *aegypti* Bites followed by Challenge with SGE

**Fig 4:**
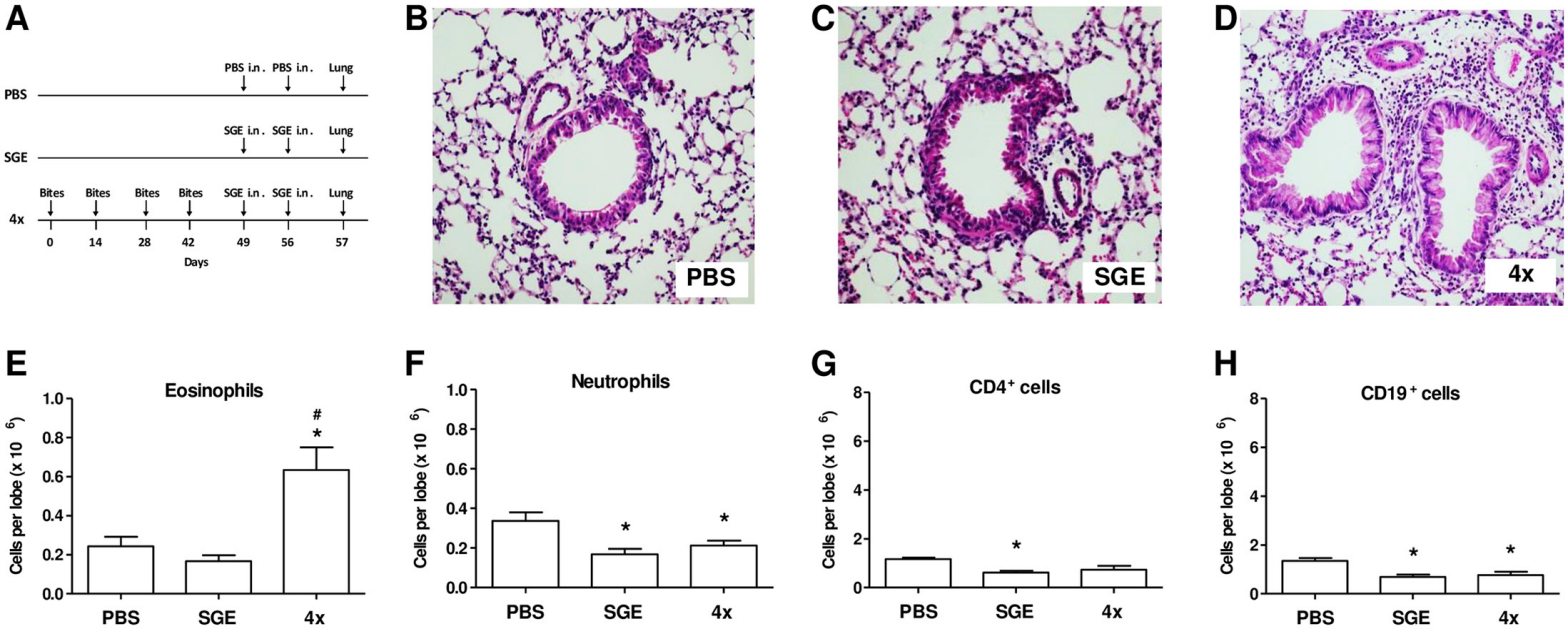
SGE challenge induces inflammation in lungs of mosquito bite-sensitized mice. Histologic examinations of lung tissue were undertaken 24 h after the last challenge with PBS or SGE. Cellular infiltration in the lungs of control and mosquito bite-sensitized groups was evaluated by H&E staining of paraformaldehyde-fixed lung tissue sections. **(A)** Sensitization protocol. Photomicrographs were taken of representative airways from each group at a magnification of 200x and are shown in **(B)** PBS; **(C)** SGE; **(D)** 4x group. After tissue digestion with collagenase and DNAse, lung inflammatory cells were separated and stained with florescence-conjugated antibodies of interest and analyzed by flow cytometry. Data represent **(E)** eosinophils; **(F)** Neutrophils; **(G)** CD4^+^ cells; **(H)** CD19^+^ cells. Results were expressed as mean ± SEM (n = 6). *p < 0.05 when compared with PBS group; ^*#*^*p* < 0.05 when compared with SGE group.

We next evaluated whether the observed changes in the BAL of mice exposed to *A*. *aegypti* bites and challenged with SGE would be reflected in their lung parenchyma (experimental protocol in Fig 4A). Histopathological analysis showed that no significant inflammatory infiltrate around the vessels and bronchus was observed in the lungs of either PBS-challenged (Fig 4B) or SGE-challenged (Fig 4C) groups. On the contrary, the 4x group presented an intense inflammatory infiltrate in the peribronchovascular area (Fig 4D). Flow cytometry immunophenotypic analysis of the lung parenchyma shows that eosinophil increase accounts for the most of the inflammatory cells present in the tissue (Fig 3E). Unlike the one observed in BAL, no significant increase was detected in number of neutrophils (Fig 4F), CD4^+^ (Fig 4G) or CD19^+^ (Fig 4H) cells in the lungs of sensitized mice. Actually, in some cases these cell populations were slightly, but significantly, decreased in the experimental groups when compared to the control group.

**Fig 5:**
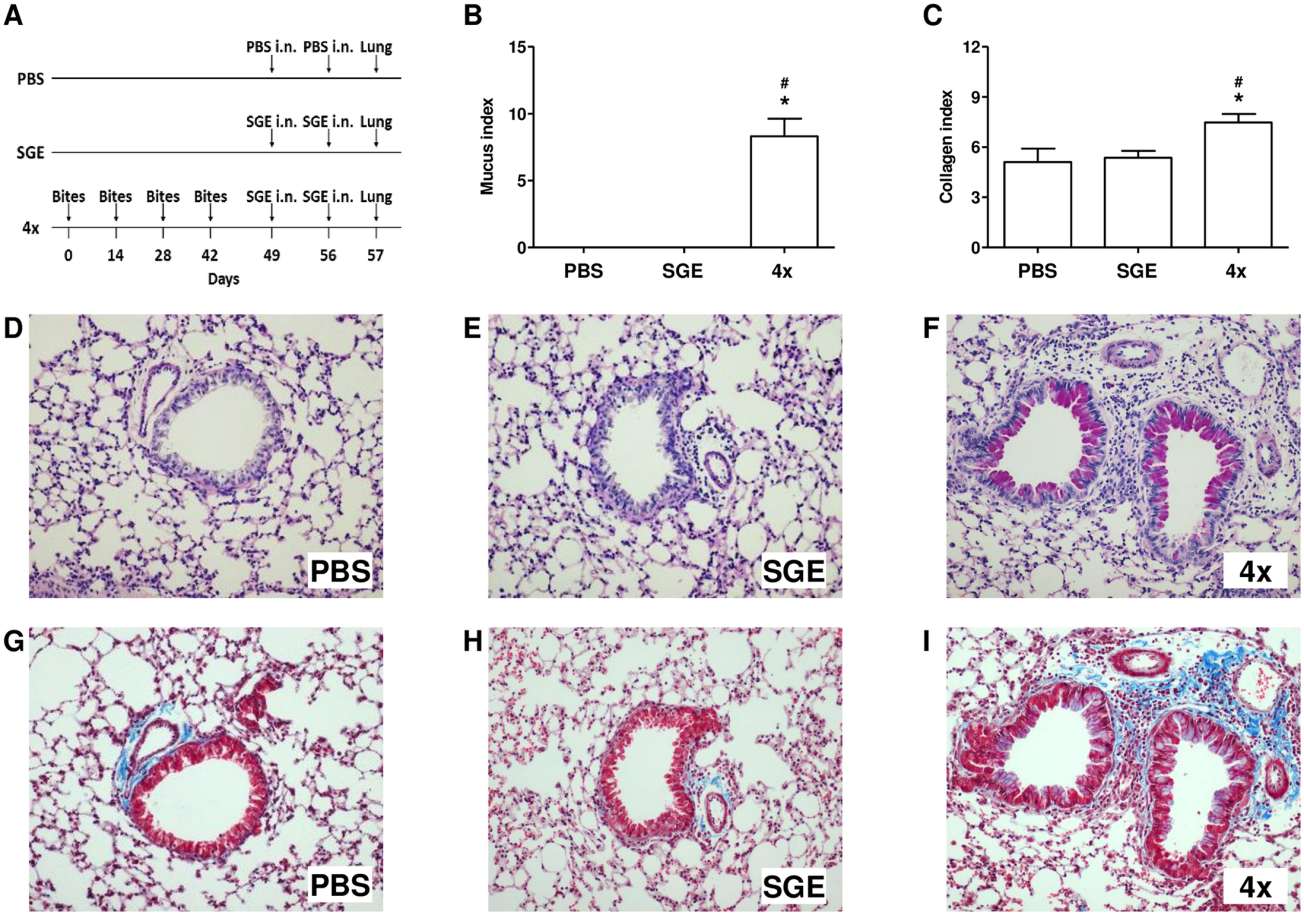
SGE challenge induces mucus production and collagen deposition in lung of mosquito bite-sensitized mice. Mucus secretion by goblet cells was evaluated in the lungs of control and mosquito bite-sensitized mice by PAS staining. Collagen deposition was evaluated in the lungs of control and mosquito bite-sensitized mice by Masson's trichrome. **(A)** Sensitization protocol; **(B and C)** morphometric analysis for mucus and collagen, respectively. Representative images from each group: **(D and G)** PBS; **(E and H)** SGE; **(F and I)** 4x group. Results were expressed as mean ± SEM (n = 6). *p < 0.05 when compared with PBS group; ^*#*^*p* < 0.05 when compared with SGE group.

Lung histological sections were evaluated to determine mucus and collagen presence in the organ (experimental protocol in Fig 5A). Representative photomicrograph of a PAS-stained lung from the PBS-challenged group (Fig 5D) shows no detectable mucus production and the same is observed in the lungs of the SGE-challenged group (Fig 5E). SGE-challenged sensitized mice (4x group) showed goblet-cell hyperplasia and mucus hypersecretion within the airway epithelia (Fig 5F). Quantitative morphometric analysis showed a large increase in mucus production in the 4x group and this increase was significantly different when compared to PBS and SGE groups (Fig 5B). Histological lung sections were stained with Masson's trichrome and the collagen deposition in peribronchovascular area was also evaluated. In the photomicrographs of PBS- (Fig 5G) and SGE- (Fig 5H) challenged groups, it is possible to observe a small collagen deposition just around the perivascular area and no collagen deposition around the bronchi. In contrast, photomicrograph of the SGE-challenged sensitized mice (4x group) presented not only collagen deposition in the perivascular region but also in the peribronchial area (Fig 5I). Morphometric analysis shows that collagen deposition observed in the lung of 4x group was significantly higher than that observed in the PBS and SGE control groups (Fig 5C).

### Exposure to *A*. *aegypti* Bites followed by Challenge with SGE Induces a Mixed-Type Antibody Production and Absence of Airway Hyperreactivity

**Fig 6:**
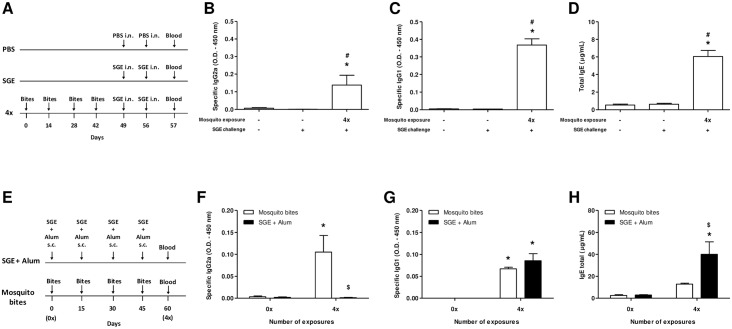
Mosquito bite-sensitization induces a mixed Th1/Th2 antibody response. Blood samples were collected from mosquito bite-sensitized mice 24 h after the last SGE challenge, and serum was separated to measure specific IgG1/IgG2a and total IgE antibodies using ELISA. **(A)** Sensitization protocol; **(B)** specific IgG2a; **(C)** specific IgG1; **(D)** total IgE. To compare the antibody response induced by naturally delivered salivary components or salivary components adsorbed in adjuvant, serum from mice exposed to mosquito bites or sensitized with SGE/Alum s.c. was collected 15 days after the last sensitization. **(E)** Sensitization protocol; **(F)** specific IgG2a; **(G)** specific IgG1; **(H)** total IgE. Results were expressed as mean ± SEM (n = 6). *p < 0.05 when compared with PBS group; ^*#*^*p* < 0.05 when compared with SGE group; ^&^*p* < 0.05 when compared with the group exposed to mosquito bites.

Blood was collected and the serum separated to measure Th1-related (IgG2a) and Th2-related (IgG1 and IgE) antibody levels (experimental protocol in Fig 6A). In non-exposed control mice challenged with PBS or SGE, no specific IgG2a or IgG1 production and very low levels of total IgE were detected. In mosquito bite-sensitized mice, an increase in specific IgG1 and total IgE antibody production was observed (Fig 6C and 6D, respectively), as expected for a Th2-related phenotype. However, specific IgG2a was also produced in these conditions (Fig 6B). Then, different sensitization strategies were carried out to compare natural exposure to mosquito bites with artificial immunization using SGE adsorbed in Alum adjuvant (Fig 6E). Under these conditions, mosquito bites again induced a mixed-type response, with high levels of specific IgG2a, IgG1 and total IgE in mice serum while SGE plus adjuvant immunization achieved a typical Th2-type response associated with barely detectable specific IgG2a, but high levels of specific IgG1 and total IgE (Fig 6F, 6G and 6H, respectively).

**Fig 7:**
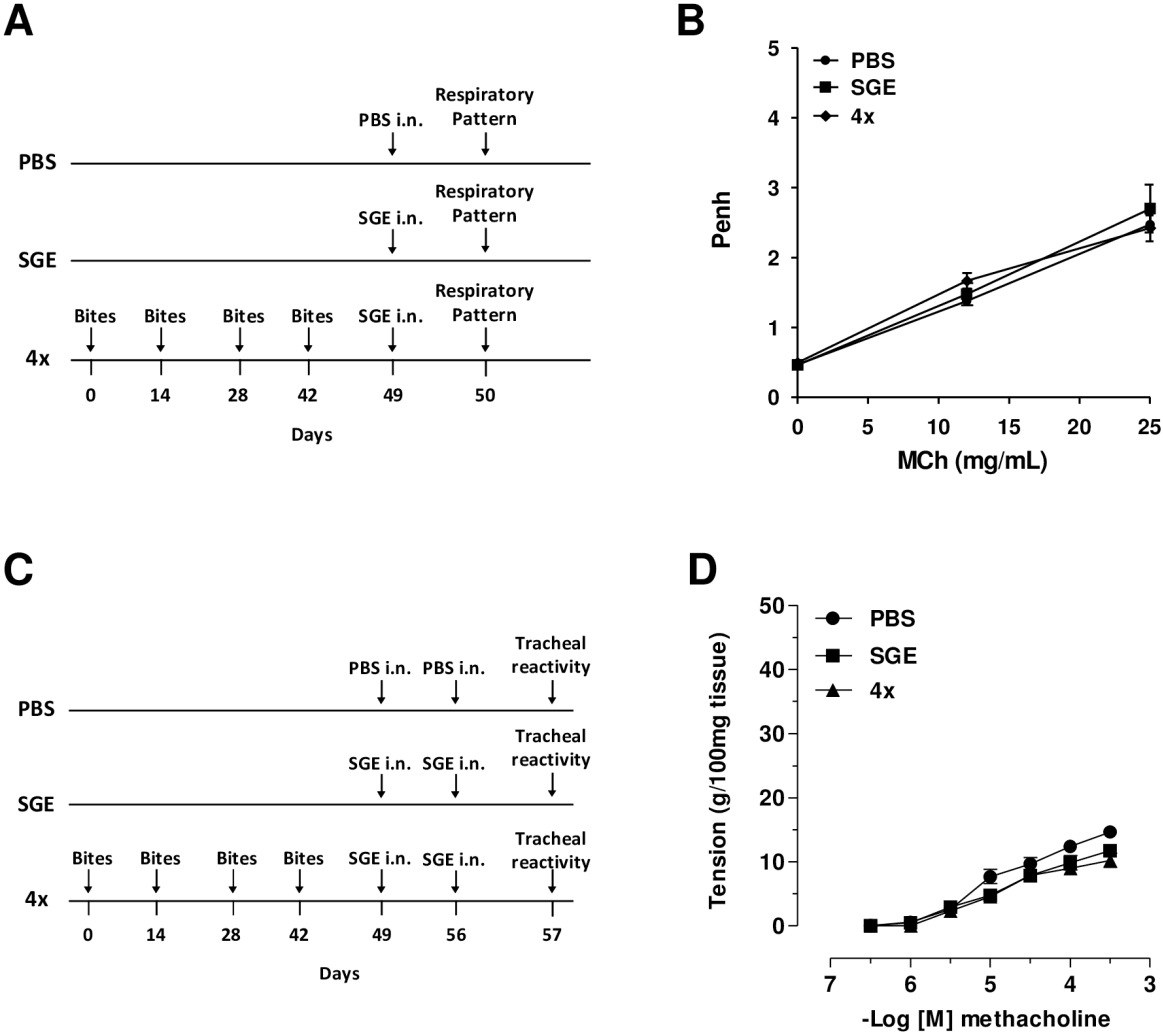
Respiratory pattern and tracheal responsiveness were not affected in SGE-challenged sensitized mice. Twenty-four hours after the last challenge, the respiratory pattern was assessed in control and mosquito bite-sensitized mice using whole-body plethysmography, as described in Material and Methods. Airway sensitivity was tested in the presence of increasing concentration of methacholine and maximal resistance values was recorded after 5 minutes. **(A)** Sensitization protocol; **(B)** results expressed as Penh. For the tracheal reactivity assay, mice were sensitized or not by mosquito bites and challenged twice with PBS or SGE. Basal values were obtained from the control group and concentration-effect curves for methacholine were constructed using an organ bath system. **(C)** Sensitization protocol; **(D)** trachea responsiveness. Results are expressed as mean ± SEM (n = 6–8). *p < 0.05 when compared with PBS group; ^#^*p* < 0.05 when compared with SGE group.

Considering all the above phenotypic features, we evaluated whether collagen deposition observed in the airways of the 4x group (Fig 5I) could be associated with changes in the lung (experimental protocol in Fig 7A). No significant differences in the breathing pattern induced by increasing doses of methacholine was observed in mice of the 4x group when compared to PBS and SGE control groups (Fig 7B). When tracheal hyperresponsiveness to methacholine was analyzed (Fig 7C), again no differences were found between the 4x group and the controls (PBS- and SGE-challenged groups) (Fig 7D).

## Discussion

Most protocols used to study allergic reactions in murine models involve the sensitization to a given antigen in the presence of an adjuvant (usually aluminum hydroxide) to induce a type 2 immune response characterized by the presence of a number of cell types and soluble mediators [24]. However, the use of adjuvants to induce allergy is an artificial method that does not correlate to the history of human disease. Even in the few studies presenting adjuvant-free antigens, the administration routes employed do not match the natural sensitization in humans [25–27]. The present work evaluated a model of natural sensitization to mosquito bites followed by an intranasal challenge with salivary antigens to investigate the parameters of allergic reaction in mice.

The cellular infiltrate to the BAL of mosquito-exposed mice following SGE challenge is in line with many experimental models of sensitization and challenge using different allergens in various mice strains [28–30]. Eosinophils are present in high numbers in many type 2-driven diseases and several models of eosinophilia have been currently used to study these cells as an important source of inflammatory and regulatory mediators such as cytokines, chemokines, lipid mediators, and cationic proteins [31–33]. In addition, proliferation and recruitment of T cells into the lungs are important to promote the pulmonary pathology observed in allergic asthmatic patients [34]. Depletion of T cells in sensitized mice inhibits asthma pathology including eosinophil recruitment to the lungs [35], and the absence of eosinophils decreases IL-4, IL-5 and IL-13 production, and also impairs T cell recruitment/accumulation of effector T cells to the lungs [34]. To date, there is no study regarding the presence of eosinophils in airways of mice sensitized by mosquito bites. On the other hand, an earlier work suggested the participation of T cells in the immune response induced by *A*. *aegypti* bites, since late responses were induced by passive transfer of spleen cells, but not serum, from mice sensitized with salivary components of *A*. *aegypti* [36]. Additionally, an intense proliferation was observed in spleen cells of sensitized mice stimulated with salivary components, suggesting that lymphocytes are involved in the development of immunological responses to mosquito saliva [37]. A significant number of B cells is also recruited to the lungs during chronic allergic lung disease but the role of this cell type in the development and maintenance of allergy remains controversial. Previous works showed that B cells have no role in the development of allergic disease [38,39] and others indicated that the involvement of these cells in allergy is primarily via IgE production [40,41].

The development of an inflammatory microenvironment observed during an allergic response allows an intense cell migration to the affected organ or tissue. In this regard, cytokines are important components and influence the progress, maintenance and exacerbation of allergic responses. It is well documented that allergen-specific Th2 responses with the subsequent release of cytokines are responsible for the cascade of events necessary for the development of an allergic response [23]. In mosquito-bitten sensitized mice, the challenge with *A*. *aegypti* SGE increased the release of IL-4, IL-5, and IL-13, but not IFN-γ or IL-17 in BAL, suggesting the development of a Th2-dominant response. IL-5 induces differentiation, proliferation, and activation of eosinophils, cooperates with eotaxins to recruit eosinophils in inflammatory conditions, increases the responsivity to eotaxin, and also primes eosinophils to respond to CCR3 ligands, the main eotaxin receptor [42–46]. Furthermore, IL-4 and IL-13 induce eotaxins, collaborating with the eosinophil recruitment [47,48]. In addition to eosinophilic infiltration, mucus production and goblet-cell hyperplasia were observed in airways of sensitized mice, a result supported by the increase in IL-13 levels observed in BAL [49,50]. Persistent inflammation by repeated allergen exposure cause several structural changes in airways. As a consequence, airway remodeling is observed in chronic asthma protocols, when the collagen deposition is higher and accompanied by mucus metaplasia of goblet cells, hyperplasia and hypertrophy of airway smooth muscle cells, excessive angiogenesis, and airway fibrosis [51,52]. We have observed collagen deposition in the peribronchovascular area of sensitized mice lungs, although not as intense as the mucus production, when compared to control mice.

Allergic airway responses are usually associated to smooth muscle hyperreactivity. Drazen *et al* [53] suggest that two distinct mechanisms can explain the hyperreactivity development: one is IL-4-, IgE-, and mast cell-dependent [54] and the other is IL-5- and eosinophil-dependent [55]. We have not observed any detectable change in the respiratory pattern or in the tracheal reactivity in response to methacholine. Keeping in mind our data for cytokine generation after SGE challenge, we suppose that none of the above mentioned mechanisms seemed to influence the airway responsiveness, evidencing an atypical allergy triggered by mosquito bites. It remains to be determined, however, whether salivary components present in the challenge could pharmacologically modify the “typical” pulmonary and tracheal reactivity.

The role of certain antibody isotypes in allergic responses is well established. IgE and IgG1 isotypes are the only ones reported as capable of inducing active and passive cutaneous anaphylaxis in mice [56,57] and, therefore, are associated to Th2 responses. IL-4 is the major differentiation factor to Th2 cells and, together with IL-13, it induces isotype switching to IgE in B cells [58,59]. The role of IgG1 depends on its origin, being anaphylactic when induced by IL-4 and non-anaphylactic when induced by IL-12 or IFN-γ [60]. Classically, allergic responses result in IgE and IgG1 antibodies and low or no IgG2a production. However, in our model of sensitization, all of these isotypes were increased in sensitized mice. These results suggest that repeated exposure to *A*. *aegypti* mosquitoes can stimulate B cells in a T-cell dependent fashion, inducing the antibody production from both Th1 and Th2 patterns. Although no increase had been seen in IFN-γ levels in the BAL, it is possible that the evaluation of this compartment was not the most appropriate to infer a systemic antibody response. In this sense, our results contrast with those observed by Chen *et al* [37] and Wang *et al* [61] employing sensitization with mosquito bites or recombinant salivary allergens without adjuvant, which induced IgE and IgG1 but not IgG2a production. However, a direct comparison with our results is not possible because they have performed different protocols of sensitization. While Chen *et al* [37] exposed each mouse to 16 female mosquitoes, twice a week for four weeks, in the present work each mouse was exposed four times to approximately 50 female mosquitoes at a 14-day interval. Moreover, Chen *et al* [37] observed a decrease in serum IgG2a levels of sensitized mice and, coincidentally, we observed the same phenomenon in animals sensitized only two times by exposure to 20 female mosquitos (unpublished data). These results suggest that a low intensity/acute exposure (20 mosquitoes/2 times) could preferentially induce antibodies of the Th2 profile, while an intense/chronic exposure (50 mosquitoes/4 times) is able to induce a mixed pattern with both Th1/Th2 antibody production. These findings corroborates classic studies showing that mosquito bites induce multiple hypersensitivity reactions depending on the degree of sensitization. Type I hypersensitivity reaction may be responsible by wheal development, while delayed papule may result from type IV hypersensitivity and immunoglobulin and complement deposits have been detected in skin suggesting that lesions may result from vasculitis induced by type III hypersensitivity [62–65]. Perhaps this explains why humans present a wheal reaction early on in the mosquito sensitization process, which disappears later on, indicating a natural process of sensitization and desensitization [66].

The clinical relevance of severe systemic allergic responses to mosquito bites has been uncovered in recent years. There are several reports of large reactions to mosquito bites, accompanied by fever, generalized malaise, hepatic dysfunction and glomerulonephritis, and Skeeter syndrome, among other symptoms [67–69]; in most cases they are mistakenly diagnosed as having infectious etiology.

## Conclusions

Taken together, our results suggest that, different from most allergy models, the exposure to mosquito bites followed by SGE challenge leads to a Th2 response in the lung environment, associated to a systemic mixed-type (Th1/Th2) reaction, and the absence of changes in the respiratory pattern and tracheal reactivity of the animals. These findings give new insights into the development of allergy to mosquito bites and can be a useful tool for the studies on allergy because they allow the evaluation of various parameters of the immune response induced by mosquito saliva, and the mechanisms involved in the processes of sensitization and desensitization to the allergen.
